# Incidence and recognition of acute respiratory distress syndrome in a UK intensive care unit

**DOI:** 10.1136/thoraxjnl-2016-208402

**Published:** 2016-08-22

**Authors:** Charlotte Summers, Nanak R Singh, Linda Worpole, Rosalind Simmonds, Judith Babar, Alison M Condliffe, Kevin E Gunning, Andrew J Johnston, Edwin R Chilvers

**Affiliations:** 1 Department of Medicine, University of Cambridge School of Clinical Medicine, Cambridge, UK; 2 John V Farman Intensive Care Unit, Addenbrooke's Hospital, Cambridge University Hospitals NHS Foundation Trust, Cambridge, UK; 3 Department of Radiology, University of Cambridge School of Clinical Medicine, Cambridge, UK

**Keywords:** ARDS

## Abstract

The reported incidence of ARDS is highly variable (2.5%–19% of intensive care unit (ICU) patients) and varies depending on study patient population used. We undertook a 6-month, prospective study to determine the incidence and outcome of ARDS in a UK adult University Hospital ICU. 344 patients were admitted during the study period, of these 43 (12.5%) were determined to have ARDS. Patients with ARDS had increased mortality at 28 days and 2 years post-diagnosis, and there was under-recognition of ARDS in both medical records and death certificattion. Our findings have implications for critical care resource planning.

The acute respiratory distress syndrome (ARDS) is characterised by pulmonary inflammation and increased pulmonary vascular permeability, which results in non-cardiogenic pulmonary oedema and refractory hypoxaemia.^1^ ARDS is associated with significant morbidity and mortality, and increased usage of critical care resources. The reported incidence of ARDS is highly variable: ARDS has been reported in 2.5%–19% of intensive care unit (ICU) patients.^2–8^ Aside from an audit conducted in 23 Scottish ICUs in 2003, which identified an ARDS incidence of 8.1%,^6^ there have been no recent studies in the UK. Reported mortality rates for ARDS also vary substantially, with values for inhospital mortality ranging from 23% to 72%.^9^ To address this we undertook a 6-month, prospective study to determine the incidence and outcome of ARDS in a UK adult University Hospital ICU.

Our study was approved by Cambridgeshire 3 Ethics Committee (08/H0306/17) and conducted in the John V Farman (general adult) ICU at Cambridge University Hospitals NHS Foundation Trust; a 20-bed ICU, serving a 1250-bed University Hospital with a local catchment of 350 000, and a wider regional referral base of ∼2 million people. A dedicated clinical research team, not involved in patient care, prospectively studied all patients admitted during a 6-month period (1 January–30 June 2009). The ICU clinical staff were not aware of the aim of the data collection. Data were collected daily until patient discharge from ICU; patients were then followed up to determine their date of hospital discharge, and survival status at 28 days, 6 months and 2 years.

All patients with a P:F ratio of <40 kPa on two arterial blood gases, taken at least 6 hours apart, and bilateral opacities on thoracic radiography had their medical notes, hospital investigations and chest radiographs reviewed by a physician who was independent of the data collection to confirm or exclude the diagnosis of ARDS.^10^ All other patients were considered not to have ARDS (‘No Lung Injury’, NLI). Assessment of left atrial hypertension was based on objective criteria when available. Patients were excluded if they had any of the following: biochemical or electrical evidence of an acute myocardial infarction, previous or current echocardiograms showing moderate or greater dilated left atrium or left ventricular dysfunction or an enlarged cardiac silhouette on a recent posterior–anterior chest radiograph. A randomly generated sample of 40% of patients with NLI had their cases reviewed in the same manner as above, to confirm the absence of ARDS. Only one case initially classified as NLI was reclassified as ARDS. For patients with a confirmed diagnosis of ARDS, copies of their hospital discharge summaries and medical coding were obtained, and the medical death certificates of those patients who died in hospital were reviewed.

Data were analysed using GraphPad Prism. Categorical variables were described using proportions and analysed using Fisher exact or χ^2^ tests. Continuous variables were described using median (IQR), and analysed using Mann-Whitney test as they were found not to follow a Gaussian distribution. Survival curves were compared using Mantel-Cox test. For the purposes of calculating length of ICU/hospital stay, the date of death was assumed to be the date of discharge from ICU/hospital. A p value of <0.05 was considered significant.

Three hundred and forty-four patients were admitted during the study period, of these 43 (12.5%) were determined to have ARDS (table 1). Our data are consistent with the previously cited Scottish study, and a multicentre European study from 2002,^6^ ^7^ suggesting that the UK may not have observed the same decline in ARDS incidence reported in US study populations.^11^

**Table 1:** Incidence and outcome of ARDS in UK teaching hospital ICU

	NLI	ARDS	p Value
n (%)	301 (87.5)	43 (12.5)	
Median age in years (IQR)	63 (49–75)	61 (45–75)	0.7690
Male, n (%)	171 (56.8%)	22 (51.1%)	0.5141
Median APACHE II score on admission to ICU (IQR)	16 (13–21)	20 (17–24)	<0.0001
Median length of hospital stay prior to ICU admission in days (IQR)	2 (1–7)	3 (2–18)	<0.001
Median length of ICU stay in days (IQR)	3 (2–6.5)	15 (4–23)	<0.0001
Median length of hospital stay in days (IQR)	23 (11–43)	38 (13–69)	0.0502
Mortality in ICU, n (%)	34 (11.3%)	18 (41.9%)	<0.0001
Mortality in hospital, n (%)	64 (21.2%)	23 (53.5%)	<0.0001
Mortality at 28 days, n (%)	46 (15.3%)	17 (39.5%)	<0.0001
Mortality at 6 months, n (%)	73 (24.3%)	24 (55.8%)	<0.0001
Mortality at 2 years, n (%)	108 (35.9%)	27 (62.8%)	<0.0001

APACHE, Acute Physiology and Chronic Health Evaluation; ARDS, acute respiratory distress syndrome; ICU, intensive care unit; NLI, no lung injury.

There was no difference in age (median 61 vs 63 years) or sex (males: 51% vs 56.8%) between patients who had ARDS and those who did not. The Acute Physiology and Chronic Health Evaluation (APACHE) II score on admission to ICU was significantly greater for patients who had or subsequently developed ARDS, compared with patients who did not (median 20 (17–24) vs 16 (13–21); p<0.0001). Pulmonary sepsis was the most common cause of ARDS, occurring in 29 out of 43 cases (67.4%). Nine cases of ARDS were associated with non-pulmonary sepsis, four cases were transfusion-associated acute lung injury (TRALI) and one occurred in the setting of polytrauma. The majority of the ARDS patient cohort (62.7%; n=27) met ARDS diagnostic criteria at the time of their admission to ICU.

Patients with ARDS had significantly fewer ventilator-free days within the first 28 days following ICU admission (median 7 (0–14) vs 27 (25–28) days; p<0.0001), and longer ICU stays than those who did not develop ARDS (median 15 (4–23) vs 3 (2–6.5) days; p<0.0001). All study patients received standard mechanical ventilation (ie, none received extracorporeal support, high frequency oscillation or other advanced respiratory support modalities). There was an increased length of hospital stay (median 38 (13–69) vs 23 (11–43) days; p=0.05) in patients with ARDS, but this was not significant at the 5% level.

Patients with ARDS had ICU and hospital mortality rates of 41.9% and 53.5%, respectively; these were significantly greater than the ICU and hospital mortality rates for patients with NLI of 11% and 21%, respectively (p<0.001, p<0.05). Figure 1 shows the Kaplan–Meier survival curves for each patient group. Patients with ARDS have a significantly reduced long-term survival compared with patients who had NLI (p<0.0001).

**Figure 1:**
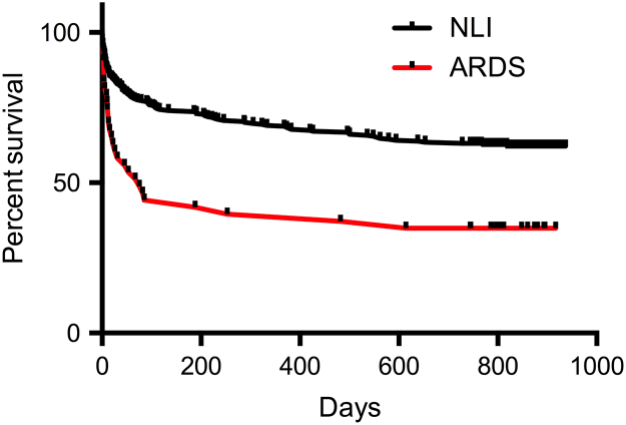
Kaplan–Meier survival curves for patients with acute respiratory distress syndrome (ARDS) or no lung injury (NLI) groups.

Despite a requirement that all patients, including those who die during their admission, admitted to our hospital should receive a discharge summary, of the 43 patients with ARDS, only 20 had a completed electronic hospital discharge summary. Remarkably, of these 20, only one made reference to the development of ARDS during the admission. Again, of the patients that died prior to hospital discharge, only one had a medical death certificate where ARDS was specified as a contributing cause of death, with many others (48%) recording ‘multiple organ failure’ as a cause of death. The lack of documentation on medical death certificates may reflect that patients often die of non-pulmonary organ failure, rather than directly from ARDS. Of the 43 patients with ARDS, only two were coded as such by the hospital's coding department.

The under-recognition of ARDS by both the clinical and coding teams, and the omission of any reference to ARDS in the medical correspondence may have wide ranging implications in terms of the immediate management for individual patients (eg, timely commencement of lung protective ventilation and other supportive measures), and in their follow-up care. Long-term follow-up studies have shown that ARDS survivors suffer major and ongoing reductions in their health/functional status.^12^ Under-recognition also has implications for resource planning with regard to ensuring critical care facilities meet demands, for example, the provision of extracorporeal membrane oxygenation services.

A limitation of this study is the potential for misclassification of ARDS, due in part to the subjective nature of the diagnostic criteria. However, to minimise this, a physician independent of the data collection team reviewed all potential cases of ARDS, as well as a representative 40% sample of patients who did not. Further weaknesses of our study include it being a single centre study, the potential for the study nurse involved in the prospective data collection to influence the outcome, and the low incidence of ARDS due to non-septic causes. Despite this, our study highlights the impact of ARDS on patient outcomes and ICU/hospital resource usage.
